# Editorial: Novel compounds from chemistry to druggable candidates

**DOI:** 10.3389/fchem.2024.1494407

**Published:** 2024-09-17

**Authors:** Pete Rose, Jawad Nasim, Yi-Zhun Zhu

**Affiliations:** ^1^ School of Bioscience, University of Nottingham, Nottingham, United Kingdom; ^2^ School of Pharmacy, Saarland University, Saarbrücken, Saarland, Germany; ^3^ School of Pharmacy, University of Macau, Taipa, Macau Region, China

**Keywords:** drugs, novel, natural products, pharmacology, bioactive

For decades natural product and novel compound research has been at the for front of drug discovery and has produced a spectrum of therapeutics that underpin modern day treatment regimes. Molecules of natural origin are often low molecular weight compounds, playing important biological functions in their host species, but once isolated or modified have value in drug discovery programmes. Other novel species that are inspired by nature have equal value in drug discovery streams especially given recent shifts towards computational chemistry and newer, greener routes of chemical synthesis. Advances in analytical chemistry applications has driven efficient molecular characterisation and isolation platforms, that when combined with genomic and biotechnological systems circumvents many of the barriers that hindered drug discovery approaches in previous decades. These developments are creating new opportunities in drug discovery research and importantly are allowing for the characterisation and isolation of novel molecules from a spectrum of differing organismal sources or synthetic libraries. The current topics “*Novel Compounds from Chemistry to Druggable Candidates*”discusses some of the recent work of colleagues in drug discovery research that are designed to identify or develop novel therapeutics. Several research articles are presented in the current topics, with additional reviews that cover antiviral compounds, marine derived sesquiterpenes, plant phytochemicals, computational chemistry and use in drug development and screening. Hopefully, the current topic issue and associated articles will facilitate interest in researchers to instigate additional drug discovery research programmes with the aim of developing future therapeutics.

In the current edition several research have contributed their valuable work that describes some of fascinating work being conducted around the world on natural products, novel compounds and drug discovery. In the review by Guo et al. the authors summarize the progress of natural products research in supporting the identification of novel antiviral agents that overcome some of the limitation and drug resistance seen over the last 2 decades. This article describes the effects of different structural types of natural products on antiviral activity thereby providing a foundation for the development of novel antiviral drugs in the future. Points of interest are descriptions of recently discovered alkaloids like isatigotindolediosides in root extracts of *Isatis indigotica*, and diterpenoids forsyqinlingine isolated from *Forsythia suspensa,* with promising antiviral properties. Also described are other molecules isolated from natural sources including examples of quinones, flavonoids and polysaccharides. In the review of Li et al. a comprehensive overview of the compound is leonurine, a molecule isolated and characterized in the tissues of *Herb leonuri* is provided. In recent years, scientists have assessed the bioactive properties of this compound that describe potent antioxidant, anti-apoptotic, and anti-inflammatory properties. Therein, are described efficient synthetic routes and isolation procedures and more recent efforts to make structural modifications of leonurine to enhance its pharmacological properties. Another fascinating field of research is in the characterisation and assessment of animal derived compounds for use in pharmacological research. Ye et al. shifts the narrative towards traditional Chinese medicines and the exploration of toad venom-derived agents (TVAs) for use in cancer research. Ye et al. reports on the various bioactivities of amphibian derived compounds and provides an overview of bufadienolides, the major bioactive components in TVAs. Descriptions of the molecular mechanisms of action provides coverage of a range of cellular targets spanning descriptions of their impacts on Na^+^/K^+^-ATPase and voltage-gated potassium channels, through to impacts on apoptotic and cell cycle pathways. In the review by Cai et al. the authors summarized recent updates in click and computational chemistry for drug discovery. Key aspects covered include development of clicking to effectively synthesize druggable candidates, synthesis and modification of natural products, targeted delivery systems, and computer-aided drug discovery for target identification, seeking out and optimizing lead compounds, and ADMET prediction. These approaches are now becoming more common place in novel compound research and with aid in optimising drug discovery streams using computational strategies. In the final review paper by Halma et al. the narrative provides an overview of novel opportunities in the development and identification of novel compounds for the inhibition of SARS-CoV-1 and SARS-CoV-2 helicases. While many studies have focused on the SARS-CoV-2 spike protein interest is also shifting to the development of replication inhibitors like, for example, the SARS-CoV-2 helicase (nsp13). This helicase shares 99.8% similarity with its SARS-CoV-1 homolog and was shown to be essential for viral replication. Halma et al. described computational studies and identified molecules that show potency to this target. These studies potentially being of interest in the anti-viral research field.

In addition to the review articles the primary research articles highlight a breadth of research in novel compound drug discovery. These articles turn attention towards the characterisation and testing of novel compounds using various chemical routes. Zhang et al. describes a genomic mining strategy to confirm the presence of genes involved in Acorane-type sesquiterpenes biosynthesis in a deep-sea derived *Penicillium bilaiae* F-28 fungus. Subsequently, 20 acorane sesquiterpenes were characterised following the large-scale fermented of fungal isolates. Importantly, of the identified molecules, 18 sesquiterpenes, namely, bilaiaeacorenols A–R were new to science. Following pharmacological assessment in an anti-inflammatory model, compound **18** exhibited the capacity to reduce NO production in LPS-induced BV-2 macrophages. These properties were dose-dependent and appeared to correlate with the capacity to inhibit LPS-induced NF-κB activation. In the article by Gao et al. the narrative shifts towards modification of the plant derived anti-malarial, artemisinin. The labile lactone structure of artemisinin is responsible for the instability of this molecule. Using strategies involving biotransformation, stains of *Cunninghamella echinulata* CGMCC 3.4879 and *Cunninghamella elegans* CGMCC 3.4832, were used to transform 10-deoxyartemisinin, a chemically modified form of artemisinin, to several novel metabolites. These products were separated and identified and tested for antimalarial activity against *Plasmodium falciparum* 3D7. This paper highlighting the novel approaches in which chemical synthesis is coupled to use of biotransformation platforms to generate novel metabolites for use in screening systems. Other articles cover refined analytical methods, computation and other *in silico* technologies to assist in drug discovery. Yu et al. focuses on plant derived compounds with a study describing the development of an UPLC-MS/MS quantification method to study the preclinical pharmacokinetics of *N*-demethylsinomenine, a potential novel analgesic candidate. Niazi et al. contributes a description of a combined synthetic chemistry and computational docking method and molecular dynamics (MD) simulation to identify small molecular modulators capable of targeting Mdm2 and Pirh2, two critical regulators of the tumour suppressor protein p53. Following screen, two synthetic lead compound MMs02943764, and MMs03738126 were found to have significant anti-proliferative effects across a range of cancer cell lines. These findings correlating with the capacity of the compounds to modulate p53 inhibitor complexes, as explored using computational platforms. Molecules were found to promote cell cycle arrest at the SubG0/G1, S, and G2 phases. This study, highlighting how multidisciplinary strategies can underpin the characterisation of novel chemicals for cancer therapy.

To summarize, this topic covers the frontiers of novel compounds in drug discovery and development. Many of the included studies raise the need for multi-disciplinary approaches that combine both synthetic or traditional ‘wet chemistry approaches’ coupled with computational or other *in silico* systems. Furthermore, these approaches are complemented by the use of robust validated biological molecules to determine compound efficacy. With the ever-rapid development of newer computation approaches, green synthetic routes, and breadth of biological screening assays, it is clear the new therapeutics will emerge in coming years using these systems.

